# Anthropogenic Eutrophication Drives Major Food Web Changes in Mwanza Gulf, Lake Victoria

**DOI:** 10.1007/s10021-024-00908-x

**Published:** 2024-05-13

**Authors:** Leighton King, Giulia Wienhues, Pavani Misra, Wojciech Tylmann, Andrea Lami, Stefano M. Bernasconi, Madalina Jaggi, Colin Courtney-Mustaphi, Moritz Muschick, Nare Ngoepe, Salome Mwaiko, Mary A. Kishe, Andrew Cohen, Oliver Heiri, Ole Seehausen, Hendrik Vogel, Martin Grosjean, Blake Matthews

**Affiliations:** 1 Department of Fish Ecology and Evolution, Swiss Federal Institute for Aquatic Science and Technology (EAWAG), Kastanienbaum, Dübendorf, Switzerland; 2 Aquatic Ecology and Evolution, Institute of Ecology and Evolution, University of Bern, Bern, Switzerland; 3 Institute of Geography and Oeschger Center for Climate Change Research, University of Bern, Bern, Switzerland; 4 Faculty of Oceanography and Geography, University of Gdańsk, Gdańsk, Poland; 5 National Research Council Water Research Institute (IRSA), Verbania, Italy; 6 Department of Earth Sciences, ETH Zürich, Zurich, Switzerland; 7 Department of Environmental Sciences, University of Basel, Basel, Switzerland; 8 Nelson Mandela African Institution of Science and Technology (NM-AIST), Arusha, Tanzania; 9 Department of Geosciences, University of Arizona, Tucson, Arizona USA; 10 Tanzania Fisheries Research Institute (TAFIRI), Dar es Salaam, Tanzania; 11 Institute of Geological Sciences and Oeschger Centre for Climate Change Research, University of Bern, Bern, Switzerland

**Keywords:** cladocera, eutrophication, food web, paleolimnology, photosynthetic pigments, tropical lake

## Abstract

Discerning ecosystem change and food web dynamics underlying anthropogenic eutrophication and the introduction of non-native species is necessary for ensuring the long-term sustainability of fisheries and lake biodiversity. Previous studies of eutrophication in Lake Victoria, eastern Africa, have focused on the loss of endemic fish biodiversity over the past several decades, but changes in the plankton communities over this same time remain unclear. To fill this gap, we examined sediment cores from a eutrophic embayment, Mwanza Gulf, to determine the timing and magnitude of changes in the phytoplankton and zooplankton assemblages over the past century. Biogeochemical proxies indicate nutrient enrichment began around ~ 1920 CE and led to rapid increases in primary production, and our analysis of photosynthetic pigments revealed three zones: pre-eutrophication (prior to 1920 CE), onset of eutrophication with increases in all pigments (1920–1990 CE), and sustained eutrophication with cyanobacterial dominance (1990 CE–present). Cladoceran remains indicate an abrupt decline in biomass in ~ 1960 CE, in response to the cumulative effects of eutrophication and lake-level rise, preceding the collapse of haplochromine cichlids in the 1980s. *Alona* and *Chydorus*, typically benthic littoral taxa, have remained at relatively low abundances since the 1960s, whereas the abundance of *Bosmina,* typically a planktonic taxon, increased in the 1990s concurrently with the biomass recovery of haplochromine cichlid fishes. Overall, our results demonstrate substantial changes over the past century in the biomass structure and taxonomic composition of Mwanza Gulf phytoplankton and zooplankton communities, providing a historical food web perspective that can help understand the recent changes and inform future resource management decisions in the Lake Victoria ecosystem.

**Supplementary Information:**

The online version contains supplementary material available at 10.1007/s10021-024-00908-x.

## Highlights

- Anthropogenic eutrophication of Mwanza Gulf began around 1920.
- Increased primary production linked to nutrient enrichment, not food web changes.
- Cladocera decline driven by eutrophication, lake-level rise, and predation pressures.

## Introduction

**Figure 1:**
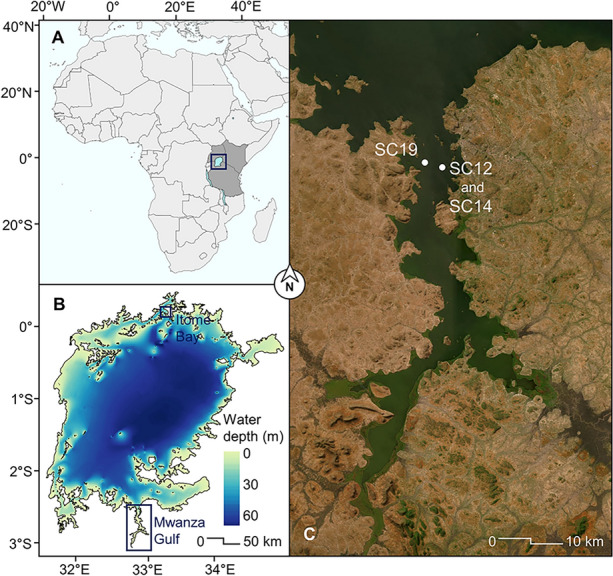
**A** The African continent with Lake Victoria. **B** Bathymetry of Lake Victoria and location of the Mwanza Gulf estuary. **C** Satellite imagery (2018–2023) of the Mwanza Gulf (Esri, Maxar, Earthstar Geographics, and the GIS User Community) with coring locations marked with white circles.

**Figure 2:**
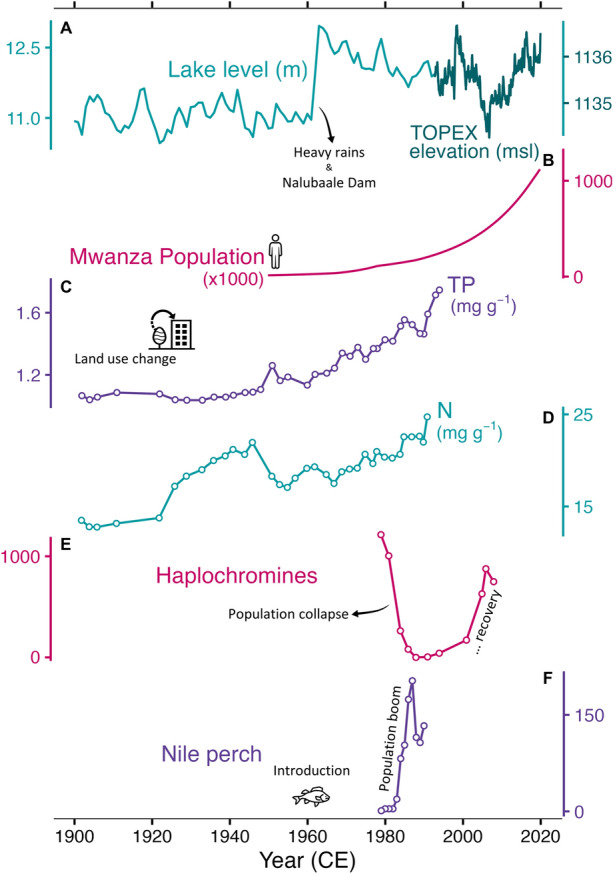
Time series data collected for Lake Victoria, including lake level monitoring (**A**; 1900–2023), the population of Mwanza City (**B**; 1950–2023), weight concentrations of nutrients (**C**–**D**; 1900–2000), and mean number of key fish taxa caught per 10 minutes of fish trawling in Mwanza Gulf (**E**–**F**; 1979–2008). Lake levels were adapted from Levêque (2017; light teal line, m above Nalubaale Dam gauge) and TOPEX satellite lake elevation observations (m above sea level, dark teal line). Population estimates of Mwanza were obtained from the United Nations World Urbanization Prospects (UN DESA 2018). Sediment extracted nutrient concentrations (TP, total phosphorus; N, total fixed nitrogen) were adapted from Hecky and others (2010) and measured from a nearshore sediment core collected from Itome Bay (Uganda). Fish survey data were obtained from Natugonza and others (2021).

Situated in one of Africa's most densely populated regions, Lake Victoria's vast resource potential has contributed to consistently higher population growth compared to the rest of the continent (Odada and others 2009). Lake Victoria (Figure 1) plays a vital role in providing ecosystem services to the local population, most notably by supporting the world's largest inland fishery (Sterner and others 2020). The lake has experienced rapid ecological change in the past century driven by various climatic and anthropogenic pressures (Figure 2), with inshore areas being particularly affected. Although long-term biological datasets are crucial for documenting such ecosystem variability, existing time series rarely span more than a few decades (Gilarranz and others 2022) and are particularly rare in tropical regions (Plisnier and others 2022). Thus, paleolimnological records serve as a complementary source of long-term data, offering insights into past environmental changes and food web dynamics in lakes (Davidson and Jeppesen 2013).

Anthropogenic eutrophication (Smith and Schindler 2009) of Lake Victoria began as early as the 1920s in response to land use change within the catchment (Verschuren and others 2002; Hecky and others 2010; Njagi and others 2022). Further complicating the observed ecological changes, higher than average rainfall and outflow damming at Jinja in the early 1960s led to a sustained 2 m increase in water level (Figure 2A). Accelerated population growth (Figure 2B) led to increased agriculture, urbanization, and deforestation, which culminated in substantial nutrient enrichment to the lake (Figure 2C,D; Hecky 1993). Subsequently, phytoplankton production increased and shifted to greater cyanobacterial dominance (Hecky 1993; Verschuren and others 2002). Increased productivity led to decreased water transparency and decreased bottom water oxygenation, which in turn impacted habitat suitability for many fish species (Kaufman 1992; Hecky and others 1994; Seehausen and others 1997).

There have been several significant changes in Lake Victoria’s fish community that have been documented over the past century, the most notable of which was the decline in species diversity and biomass of haplochromine cichlid fishes (Figure 2E) and the introduction and population expansion of Nile perch (*Lates nilotocus*, Figure 2F). Fisheries records from the 1960s (compiled in Figure 2E) suggest that endemic haplochromine cichlids constituted > 80% of total fish biomass in Mwanza Gulf (Kudhongania and Cordone 1974), and this biomass constituted more than 120 different species in the Mwanza Gulf alone (Witte and others 1992). However, there was a major decrease in haplochromine biomass and diversity in the 1980–1990 s, which has several possible underlying causes, including (i) reduced habitat availability, particularly in the shallower inshore gulfs where the effects of eutrophication have been more intense (Mugidde 1993), (ii) intensive fishing pressures (Witte and others 1992; van Zwieten and others 2016), and (iii) the introduction and later population explosion of Nile perch (a large piscivorous predator) along with the early loss of piscivorous haplochromines that feed on juvenile Nile perch (Witte and others 2007). Another major change in the fish community starting in the 1980s was the population increase of the native cyprinid, dagaa (*Rastrineobola argentea*, a small zooplanktivore), which may have occurred in response to reduced competition from the declining haplochromine cichlid biomass and the loss of predatory haplochromines (Wanink 1999; Goldschmidt and Witte 1992). Some recovery of the haplochromine cichlid biomass has been observed in the past decades (Figure 2E), but much of the species diversity remains lost (Witte and others 2000; Kishe-Machumu and others 2015).

Despite intensive research on Lake Victoria’s fish community in the past few decades (Kolding and others 2014; van Zwieten and others 2016), there are limited empirical data to provide insight into changes in the phytoplankton and zooplankton communities over the past century. In the absence of lake monitoring data, analysis of lake sediments using photosynthetic pigment biomarker concentrations (Leavitt and Hodgson 2001) and subfossil remains of Cladocera and *Chaoborus* aquatic insect larvae (Korhola and Rautio 2001; Verschuren and others 2002) can provide insights into past phytoplankton and zooplankton community structure (that is, abundance and taxonomic composition). For example, such reconstructions can be useful for identifying how the timing of changes in plankton community structure relate to changes in lake productivity and fish community structure (Skov and others 2010).

To elucidate the impact of anthropogenic eutrophication on food web dynamics of Lake Victoria, we examined a wide range of paleolimnological indicators from two coring sites located in the Mwanza Gulf (Figure 1). We used multiple biogeochemical proxies to provide insight into potential changes in nutrient availability. Sedimentary photosynthetic pigments were measured as an indicator of past phytoplankton community composition and total algal biomass. Lastly, zooplankton community structure was explored using sedimentary cladoceran subfossils, and related to survey records of fish abundance. Our main objectives were to: (1) investigate the onset of anthropogenic eutrophication and associated ecological changes in the Mwanza Gulf over the past century and (2) examine the temporal changes in the zooplankton and zoobenthos assemblage in relation to changes in the abundance and composition of primary producers, as well as fish community structure (Figure 2E,F).

## Materials and Methods

### Study Site Description

Located in equatorial eastern Africa, Lake Victoria (Figure 1) is the world's largest tropical lake (surface area = 68,800 km^2^, mean depth = 40 m). The lake is well known as a biodiversity hotspot, featuring a prolific fish community of over 500 haplochromine cichlid species (Genner and others 2004). Previous paleolimnological research has investigated eutrophication in northern Lake Victoria (Verschuren and others 2002; Hecky and others 2010; Njagi and others 2022), but its impact in southern inshore areas of Lake Victoria remains understudied. Specifically, the Mwanza Gulf (Figure 1; 60 km long, 2.5–11 km wide) is the largest Tanzanian port on Lake Victoria and has previously been recognized for receiving the highest daily municipal water pollution within Tanzania (Juma and others 2014). The composition and abundance of phytoplankton in Mwanza Gulf vary from that observed in the open water and northern gulfs (Frank and others 2023). The surrounding land is primarily agricultural (> 60%) and urban (~ 16%), with less than ~ 5% remaining as unconverted wetlands and woodland (Cornelissen and others 2014). Non-native Nile perch and water hyacinth (*Eichhornia crassipes*, a free-floating macrophyte) were first reported in the Mwanza Gulf in 1961 (Pringle 2005) and 1990 (Witte and others 1995), respectively. However, the Nile perch population remained low until the early 1980s when the population increased exponentially (Witte and others 1992).

### Sediment Core Collection and Subsampling

Sediment cores were collected in 2018 from two sites in the Mwanza Gulf using a UWITEC gravity corer (60 mm internal diameter; Figure 1c). Two of the cores were collected as a paired set (SC12 and SC14, length = 37 and 28 cm, respectively) at the same coring location (2° 33.473′ S, 32° 52.470′ E) with a water depth of 14.5 m, whereas the third core (SC19, length = 47 cm) was collected on the opposite side of the gulf (2° 33.015′ S, 32° 51.023′ E) at a water depth of 10.5 m. Cores were split lengthwise, and the core face was scanned using hyperspectral imaging (HSI) and x-ray fluorescence (XRF) techniques. Core halves were wet subsampled contiguously in 1–2-cm intervals depending on the required sediment volume for the analysis.

### Geochronological Dating

Sediment samples from the cores SC12 and SC19 were analyzed for ^137^Cs and ^226^Ra using gamma-spectrometry and for ^210^Pb (via^210^Po) by alpha-spectrometry. ^226^Ra measurements were difficult due to small sample mass (0.4–0.8 g) and provided unstable results; thus, constant supported ^210^Pb activities were calculated from the mean for the lowermost parts of the core profiles. Sediment ages were modeled using the Bayesian *plum* model (Table S1, Table S2; Aquino-López and others 2018). Additionally, we tested the sensitivity of the age-depth model choice by comparing the *plum* ages with the Constant Flux Constant Sedimentation (CFCS) and Constant Rate of Supply (CRS) age models (Figure S1; Appleby and Oldfield 1978). SC14 was stratigraphically correlated with the SC12 chronology based on hyperspectral-inferred total chloropigment (TChl) profiles, with linear interpolation used to assign dates between correlation points (Figure S2).

### Biogeochemical Indicators

Non-destructive biogeochemical methods included scanning the surface of split core halves with HSI and XRF techniques. HSI was used to examine changes in total chloropigments in the sediment (TChl: Chlorophyll *a, b,* and derivatives; Butz and others, 2015). All cores were scanned with a Specim Single Core Scanner system (Spectral Imaging Ltd., Oulu, Finland) equipped with a Specim PFD-xx-V10E camera (400–1000 nm). Relative absorption band depth index (RABD_655-680_) was used to quantify total chlorophylls and colored derivatives.

X-ray fluorescence (XRF) was carried out to assess changes in the elemental composition of sediments indicative of major shifts in the lake environment. Scans were performed using an ITRAX (Cox Ltd., Sweden) with a chromium anode at 50 mA, 30 kV, and 30 s integration time over 0.5 cm intervals.

Sequential phosphorus (P) extraction protocol for SC12 and SC19 followed the protocol developed by the Standards, Measurements, and Testing (SMT) program (Ruban and others 2001) with modifications following Tu and others (2021). Three independent extractions using NaOH and HCl were completed to measure five P fractions: non-apatite inorganic P (hereafter referred to as Fe–P), calcium phosphate apatite (AP), inorganic P (IP), organic P (OP), and total P (TP). P concentrations in unfiltered samples were measured spectrophotometrically (Shimadzu UV-1800) with the malachite green method at an absorbance of 610 nm (Ohno and Zibilske 1991).

Total carbon and nitrogen isotope analyses (TC%, TN %, δ^13^C, δ^15^N) were undertaken to evaluate shifts in trophic state conditions through time. Sediment subsamples were freeze-dried, homogenized, and weighed into tin capsules. Samples were then measured via combustion using a ThermoFisher Flash-EA 1112 coupled with a Conflo IV interface to a ThermoFisher DeltaV isotope ratio mass spectrometer. Isotopic compositions are reported in conventional delta notation relative to the international standards (Vienna Pee Dee Belemnite (V-PDB) and atmospheric N_2 (AIR)_). Signatures of δ^13^C were corrected for the Suess effect (Figure S3) following Verburg (2007). TC corresponds to total organic carbon as prior tests indicated inorganic carbon was absent in sediment samples.

### Photosynthetic Pigments

Sedimentary pigments were extracted from ~ 200 mg dry homogenized sediment following Sanchini and Grosjean (2020). Extracts were quantified using the methodology of Lami and others (1994, 2000) by high-performance liquid chromatography (HPLC). Analysis was restricted to taxonomically diagnostic pigments (Table 1). The chlorophyll *a* preservation index (CPI) was calculated as the ratio of Chl *a* to the sum of Chl *a* and derivatives, with low values indicating poor preservation (Buchaca and Catalan 2007).

**Table 1:** Biological Indicators Used and Their First-Order Interpretation

Trophic level	Indicator	Taxonomic affiliation and ecological interpretation
Phytoplankton (photosynthetic pigments)	*β,β*-carotene	All primary producers
	Chlorophyll *a*	All primary producers (sum of isomers)
	Pheophytin *a*	Chl *a* derivative (sum of isomers)
	Pheophorbide *a*	Chl *a* derivative (sum of isomers)
	Alloxanthin	Cryptophytes
	Diatoxanthin	Diatoms
	Diadinoxanthin	Diatoms, dinoflagellates, chrysophytes
	Dinoxanthin	Dinoflagellates & chrysophytes
	Peridinin	Dinoflagellates
	Lutein	Chlorophytes
	Echinenone	Total cyanobacteria
	Canthaxanthin	Colonial cyanobacteria
	Myxoxanthophyll^*^	Cyanobacteria
	Oscillaxanthin	Cyanobacteria (Oscillatoria)
	Zeaxanthin	Carotenoid characteristic of cyanobacteria
Zooplankton (subfossils)	Cladocera: *Alona*	Littoral, scraper feeders (Fryer 1968)
	*Chydorus*	Littoral, scraper feeders (Fryer 1968)
	*Bosmina*	Planktonic, selective feeders (Korhola and Rautio 2001)
	Diptera: *Chaoborus*	Planktonic larvae with diel vertical migration, predatory (Dawidowicz and others 1990)

Pigment affiliations are based on Leavitt and Hodgson (2001), Buchaca and Catalan (2007), and Lami and others (2009). Asterisk indicates only detected in degraded form.

### Zooplankton Subfossils

The concentration of zooplankton subfossils (calculated as the number of individuals per volume of wet sediment) was determined from SC14 subsamples. The volume of sediment for each subsample was measured, and subsamples were then wet sieved through a 38-µm mesh. *Lycopodium clavatum* marker spores (mean number of spores = 9666, *σ* = 671; Lund University, Batch 3862) were dissolved in each subsample to assess the proportion of sediment examined. While ensuring the sample was adequately mixed, the solution was permanently mounted on glass slides and each slide was examined across its entirety using bright-field illumination on a compound microscope at 200–400x. Cladoceran remains were identified to the most detailed taxonomic level possible (based on Szeroczyńska and Sarmaja-Korjonen 2007; King and others 2024a, 2024b) and counted separately. The minimum number of individuals was determined by the most abundant body part of each taxon (Zharov and others 2022). Concentrations were calculated by dividing the minimum number of individuals by the volume of sediment screened (determined by multiplying the total subsample volume by the proportion of marker spores counted relative to the total number of marker spores added). The influx of individuals to the sediment was then calculated by dividing the concentrations by the number of years per cm of depth.

### Statistical Analyses

Stratigraphically constrained cluster analysis (CONISS; Grimm 1987) was performed on the SC12 pigment and SC14 cladoceran datasets to identify the timing of shifts between distinct assemblages. Prior to clustering, pigment and cladoceran concentrations were log-transformed (pigments only) and scaled to a mean of 0. Zone determination used a broken-stick model (Bennett 1996). SC19 pigments were not clustered due to deeper sediment mixing. A modified randomized intervention analysis (RIA; Carpenter and others 1989), which excluded the calculation of interecosystem differences, was performed on the individual pigments and zooplankton taxa to assess the significance of observed changes between pre- and post- intervention data. Intervention points at 14, 18, or 26 cm for SC14, SC12, and SC19, respectively, were based on the geochronological layers for 1920 CE (Common Era), which represented the onset of eutrophication determined from pigment clustering. For the Cladocera taxa, we proceeded using the onset of eutrophication rather than the changepoint identified by CONISS as we cannot exclude the possibility of cumulative effects between eutrophication and other events. The observed change was calculated as the difference in mean before and after the intervention. The test statistic was then derived by performing one thousand random permutations of each time series and calculating the distribution of the mean difference before and after 1920 for each metric (R Core Team 2022, version 4.2.2). Significance levels (*p*-values) were calculated as the proportion of randomized mean differences equal to or exceeding the observed intervention effect in absolute value.

## Results

### Geochronology

**Figure 3:**
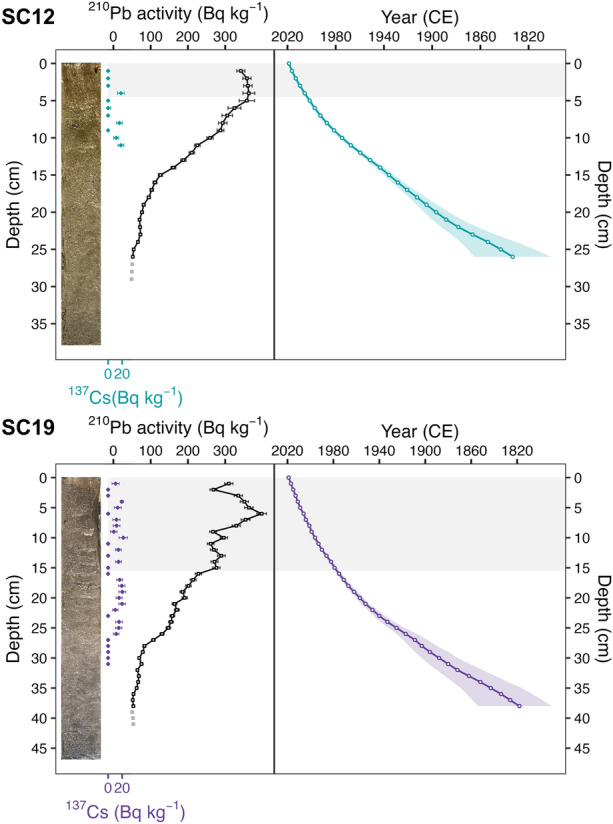
Total ^210^Pb (black open squares) and ^137^Cs (diamonds) specific activity with error bars, measured throughout SC12 (teal) and SC19 (purple). The lowermost parts of the profiles indicate the supported ^210^Pb (gray filled squares). Age-depth models were calculated for SC12 and SC19 based on the Bayesian *plum* model (Aquino-López and others 2020; 95% confidence interval indicated by shaded ribbon). Gray shading indicates core layers with turbated sediment.

Total ^210^Pb was observed to decline downcore in both cores, reaching supported ^210^Pb levels at 25 cm (SC12) and 35 cm (SC19), respectively (Figure 2). Sediment disturbance and/or bioturbation was inferred from increasing downcore ^210^Pb specific activities in the uppermost 4 cm of SC12 and 6 cm of SC19, which deviate from the ideal exponential decline expected for unsupported ^210^Pb. Additionally, the section from 10 to 15 cm in SC19 showed a lack of ^210^Pb decay, which indicates deeper sediment disturbances due to shallower water depth or the higher potential of trawling at this site (Witte and others 2012). Alternatively, increasing sedimentation rates may have compensated for the radioactive decay of ^210^Pb. The majority of our subsequent analyses focused on SC12 due to the lesser degree of sediment disturbance. Age-depth models extended back to ~ 1900 CE (± 9 years), with age estimates before this year having large uncertainties (Figure 2). Given the relative agreement between modeling approaches (Figure S1), we proceeded using the Bayesian *plum* model as it provides more realistic conservative estimates for deeper sediments (Aquino-López and others 2018; Hunter and others 2023). This model aligns well with the range of the most likely age-depth distribution. Concentrations of ^137^Cs in the sediments were not sufficient to constrain either core chronology, which is typical for tropical African lakes (Walling and He 2000). Thus, downcore ^137^Cs concentrations (Figure 3) were attributed to post-depositional diffusion through pore waters (Klaminder and others 2012) or sediment mixing.

### Biogeochemical and Isotopic Sediment Composition Over Time

**Figure 4:**
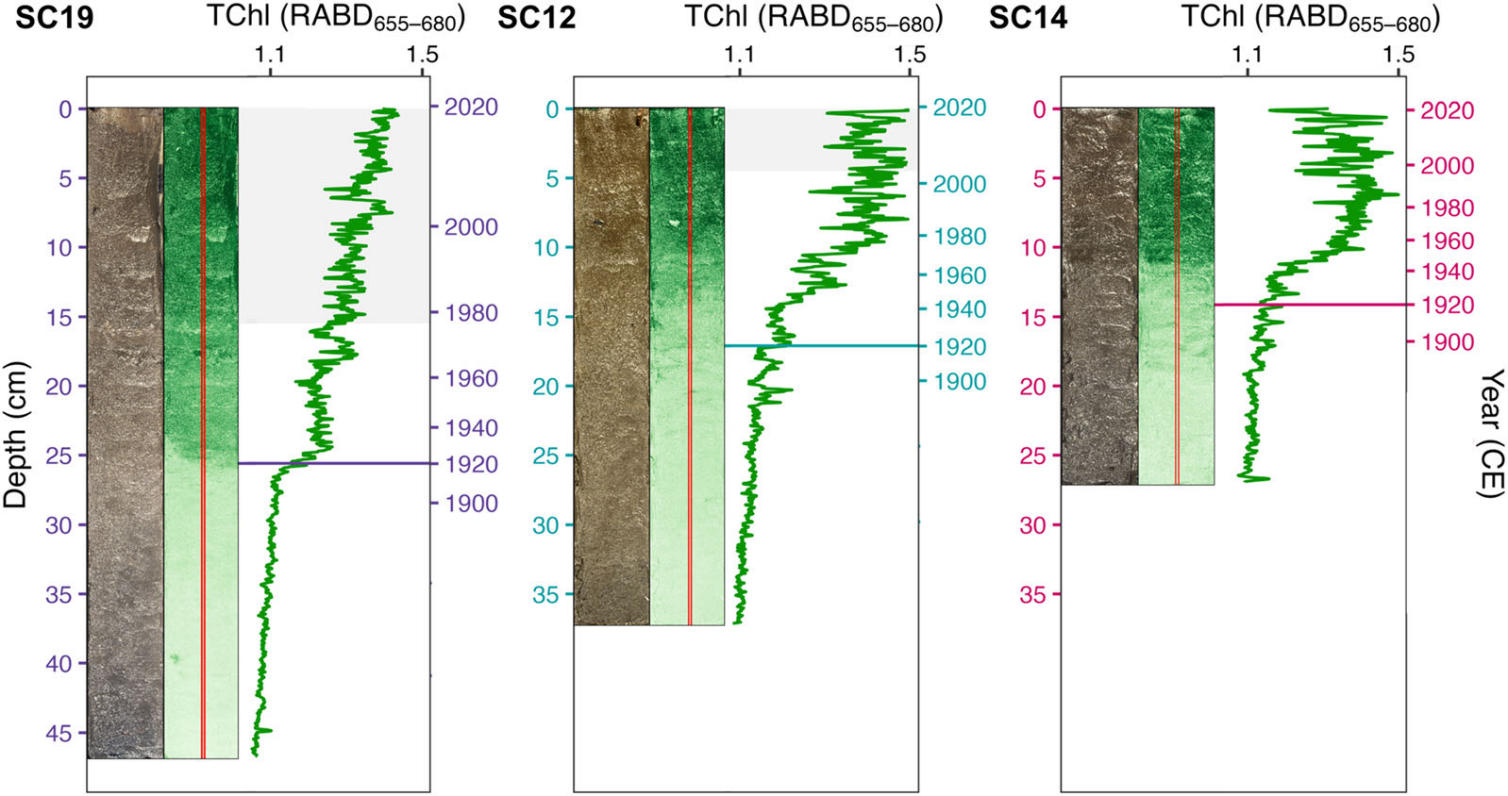
Color photographs of split core faces, colorized image for chloropigment stratigraphy and total chloropigment (TChl) moving average (*k* = 13 samples) profiles of SC19 (purple), SC12 (teal), and SC14 (pink). Solid horizontal lines indicate the year 1920 CE (± 7 years, 95% CI) based on the chronology of each core, and gray shading indicates core parts with turbated sediment.

**Figure 5:**
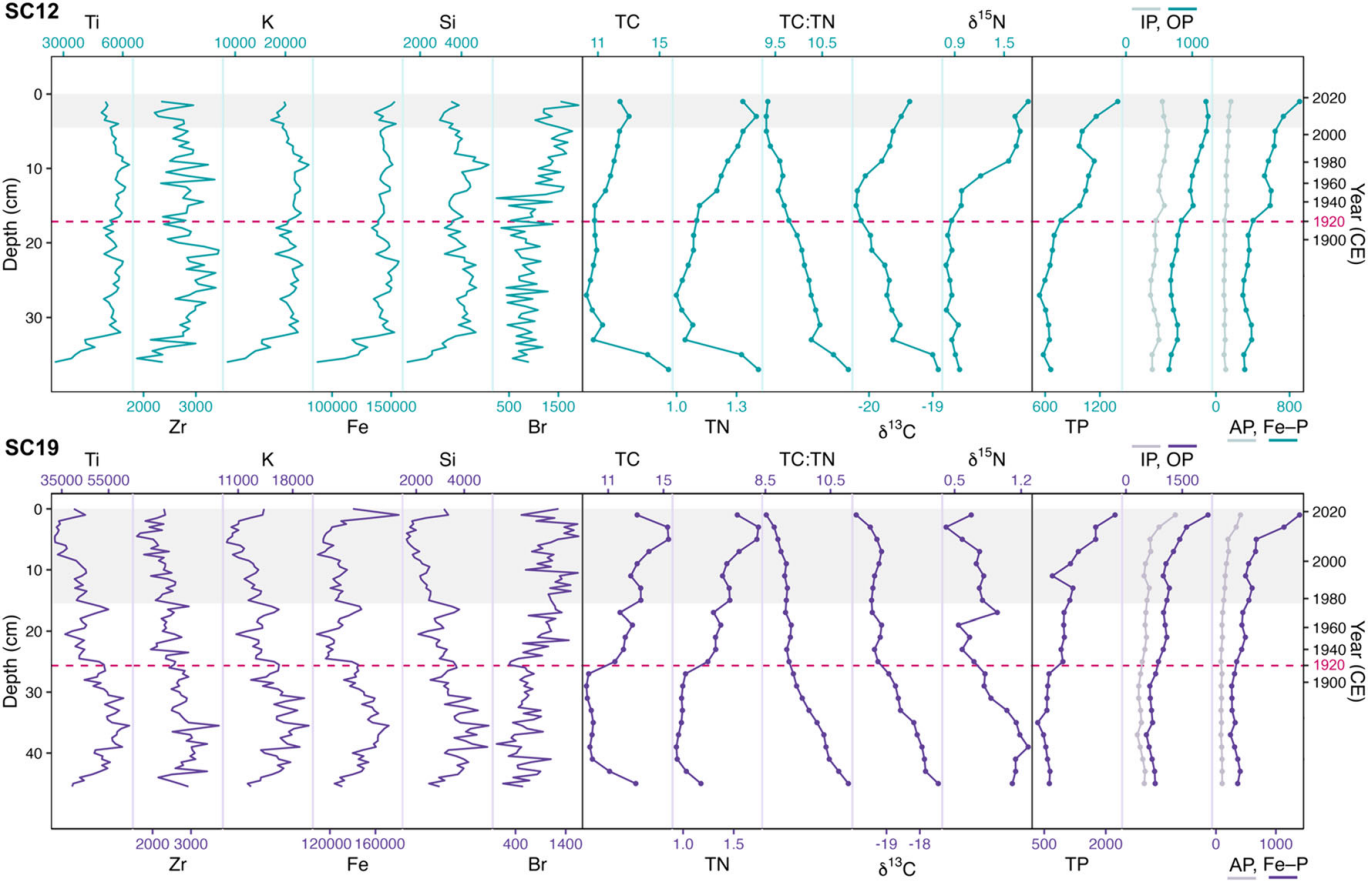
Biogeochemical proxies analyzed in SC12 (teal) and SC19 (purple), including: XRF (Ti, Zr, K, Si, Br; total counts), carbon and nitrogen isotope geochemistry (TC, %; TN, %; C:N; Suess-corrected δ^13^C, ‰; δ^15^N, ‰), and phosphorus concentrations (TP, IP, OP, AP, Fe–P; µg P g^−1^_d.s._). Dashed horizontal lines indicate the year 1920 (± 7 years, 95% CI), and gray shading indicates core parts with turbated sediment.

Mwanza Gulf cores showed a consistent pattern of nutrient enrichment and increased productivity over the past century. TChl index values remained low until the 1920s, followed by a rapid increase until peaking around 1985 and subsequently stabilizing (Figure 4). Values of lithogenic material (Ti, Zr, K, Fe, Si) remained relatively stable over the past century, while organic material (Br) increased after 1920 CE (Figure 5). Atomic ratios of TC:TN (range = 9.3–11.0) exhibited decreasing trends in both cores over the past century (Figure 5). Values of δ^15^N displayed minor changes across both cores (range = 0.4–1.3‰). Suess-corrected δ^13^C values decreased gradually until the early-1960s in both cores and subsequently exhibited differing trends with further decreases observed in SC19 and increased values in SC12 (Figure 5). All P fractions exhibited increasing trends over time. Among these fractions, OP and Fe–P demonstrated the most substantial rises, significantly contributing to the overall TP increase. TP concentrations in the sediments ranged from 538.2 to 1395.9 µg/g_d.s._ and 340.8 to 2225.5 µg/g_d.s_ in SC12 and SC19, respectively.

### Food Web Responses to Eutrophication (Photosynthetic Pigments and Zooplankton)

**Figure 6:**
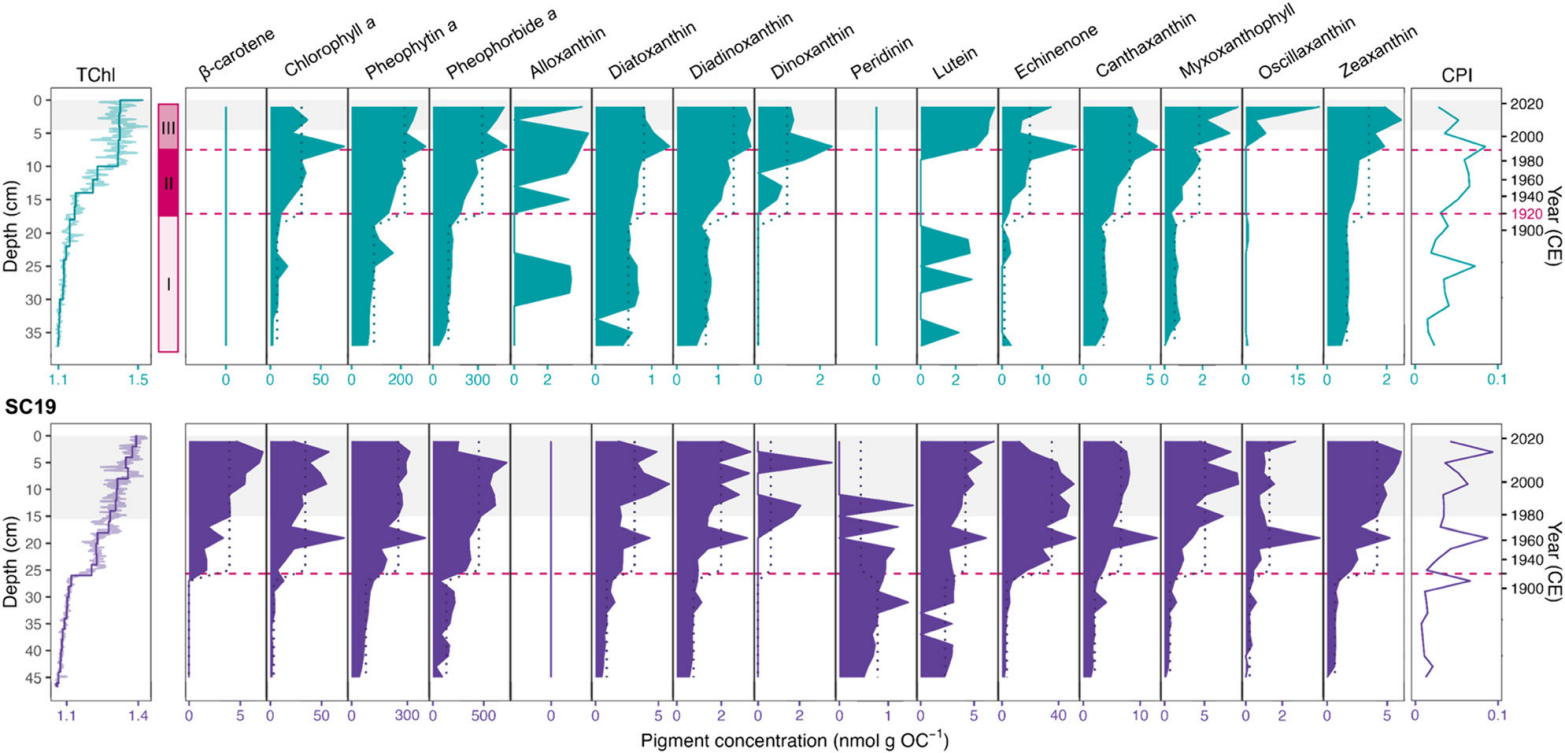
Binned (2 cm intervals) hyperspectral total chloropigments (TChl), relative concentrations of photosynthetic pigments (nmol g OC^−1^), and chlorophyll preservation index (CPI) measured throughout SC12 and SC19. Zone (I–III) differentiation was based on cluster analysis. Dashed horizontal lines indicate 1920 (± 7 years, 95% CI) and 1990 (± 2 years), and gray shading indicates core parts with turbated sediment. Dotted lines indicate significant differences between means before and after 1920. Pigment associations are listed in Table 1.

Photosynthetic pigments displayed a trend of increasingly elevated relative concentrations following the onset of eutrophication, regardless of increasing flux after ~ 1986 CE (Figure 6; Figure S4). Most pigments were detected in both SC12 and SC19, with the exception of *β,β*-carotene and peridinin in SC12, and alloxanthin in SC19. CONISS revealed three zones consisting of distinct pigment assemblages: pre-eutrophication (prior to 1920 CE), onset of eutrophication with increases in most pigments (1920–1990 CE), and sustained eutrophication with cyanobacterial dominance (1990 CE–present). RIA indicated mean concentrations increased to values ~ 2–10 × times higher than the low stable concentrations observed prior to 1920 (Figure S5, S6, Table S3).

**Figure 7:**
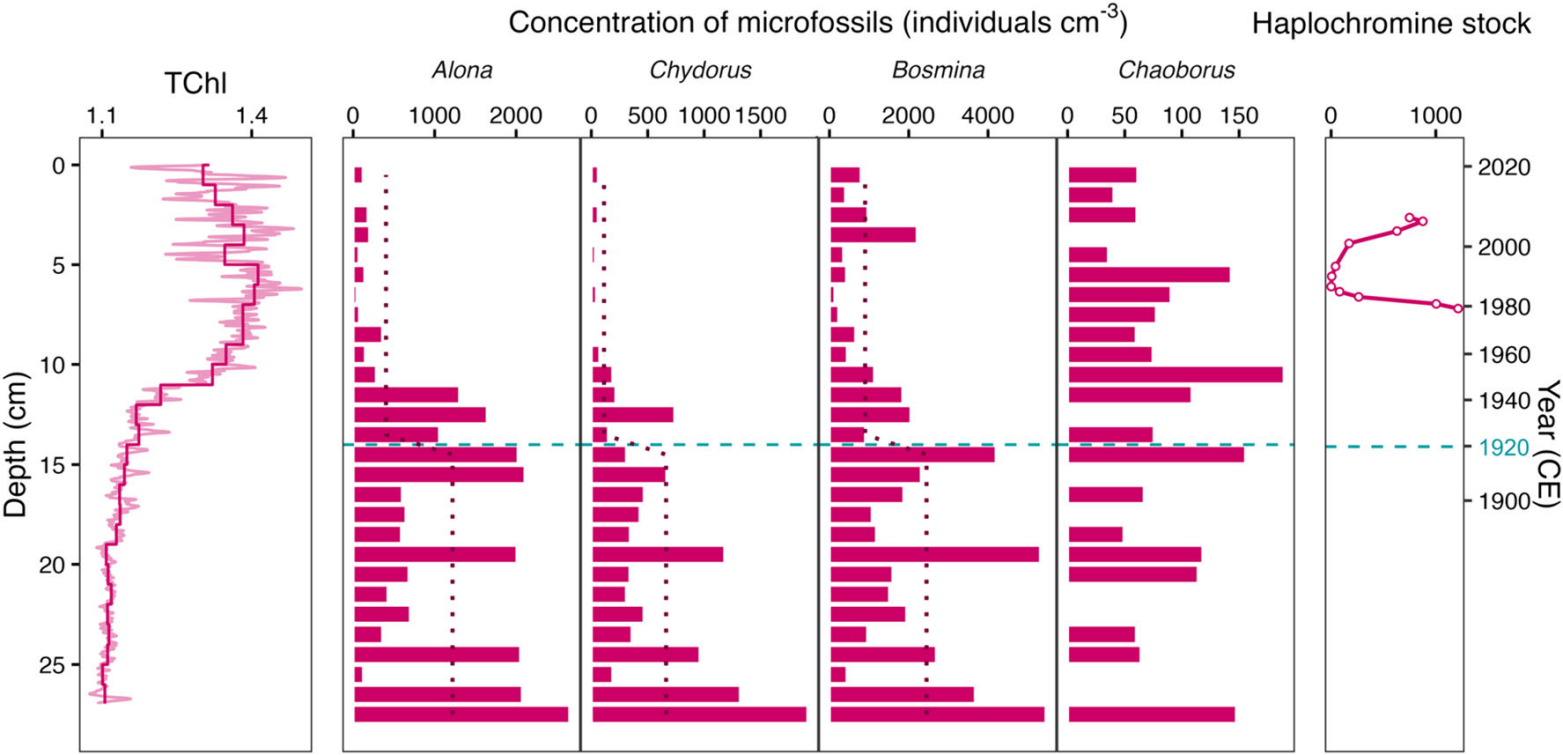
Binned (1-cm intervals) hyperspectral total chloropigments (TChl) and concentration of zooplankton subfossils counted throughout SC14, as well as mean number of haplochromines caught per 10 minutes of trawling (see Natugonza and others 2021). Dashed horizontal line indicates 1920 CE (± 7 years, 95% CI). Dotted lines indicate significant differences between means before and after 1920.

The zooplankton of SC14 consisted of three genera of cladoceran taxa, including benthic chydorids (*Alona* and *Chydorus*) and planktonic *Bosmina*, as well as *Chaoborus* mandibles (Figure 7). All cladoceran taxa exhibited steadily high concentrations until the early-1900s followed by a major decrease around ~ 1957 (± 5 years, Figure S7) despite consistent sampling effort (Figure S8) and changes in sedimentation rates (Figure S9). RIA suggested a significant change in each cladoceran genus after 1920, exhibiting decreased mean concentrations by ~ 60–85% (Figure 7, S10; Table S4). Notably, *Bosmina* remains were consistently present with a subsequent increase, whereas *Alona* and *Chydorus* remain at low abundances. *Chaoborus* mandibles were rarely encountered, with at most two individuals being found per subsample (Figure S7); thus, no significant change was detected by RIA.

## Discussion

Our results suggest that anthropogenic eutrophication of Lake Victoria and the increase in lake level (Figure 2) have had major impacts in the plankton communities of Mwanza Gulf. Nutrient enrichment (P and N) began ~ 1920 CE at Mwanza Gulf and rapidly caused increased primary production. Analysis of photosynthetic pigments revealed three stratigraphic zones, including pre-eutrophication (prior to 1920), the onset of eutrophication (1920–1990), and sustained eutrophication (1990–present), that featured increasingly higher pigment concentrations. The cladoceran assemblage, particularly the benthic groups, collapsed in the late-1950s/early-1960s and then partially recovered following the recovery of the haplochromine cichlid population in the 1990s. The timing of the decline is most likely attributable to rising water levels and reduced resource quality that resulted from eutrophication.

### Reconstructing Anthropogenic Eutrophication

Nutrient enrichment and excessive algal growth were evident in Mwanza Gulf, marked by a rapid increase in nutrients (P) and productivity (TChl) around the core depths representative of ~ 1920 (Figure 4). Biostratigraphical analysis of SC12 photosynthetic pigments identified a discernible shift in assemblage at ~ 16–18 cm, corresponding with ~ 1920 (± 15 years). While the 1920 timing of N enrichment is consistent with previous observations of surface cores, enhanced P deposition began earlier in Mwanza Gulf compared to northern inshore areas in which it only began to increase in the 1940s (Hecky and others 2010). TP and bioavailable P (OP and Fe–P) concentrations exhibited an increasing trend since ~ 1920, contrasting with low stable values of AP (Figure 5). Further increases in TP until present are consistent with estimates indicating a > 100% increase in surface water TP concentrations between the 1960s and 1990s (Hecky and others 2010). The stability of AP (non-bioavailable in sediments; Tu and others 2021) and lithogenic material (Ti, Zr, K, Fe) suggests that changes in organic sediment components (for example, OP and Br) are likely driven by increased primary productivity and autochthonous organic matter deposition rather than detrital input. Altogether, this indicates an earlier onset of eutrophication in Mwanza Gulf compared to northern Lake Victoria and highlights the contextual nature of spatial and temporal lake ecosystem responses to anthropogenic eutrophication.

Primary production in the Mwanza Gulf reached unprecedented levels in the history of the modern lake in the ~ 1980–1990 s and subsequently stabilized (Frank and others 2023). Analysis of bulk organic matter using TC:TN ratios revealed steady declines (Figure 5) consistent with increasing algal dominance. High ratios (> 20) are typically indicative of vascular land plants, while lower ratios (5–8) represent plankton dominance (Meyers 1994; Finlay and Kendall 2007). Thus, declining ratios are typical of lakes experiencing shifts to turbid, phytoplankton-dominated conditions (for example, King and others 2024a, 2024b). Despite its high cellulose content and rapid growth rate, the expansion of water hyacinth cover in 1990 is not reflected in the TC:TN ratio, which attests to the success of the rapid application of biological control agents and awareness campaigns (*Neochetina* weevils; Wilson and others 2007). TC:TN ratios of Mwanza Gulf were well within the range (typically 8.3–14.6) indicative of moderate N-limitation (Hecky and others 1993), which is consistent with previous reports of Lake Victoria (Talbot and Lærdal 2000). At SC12, the reversal of the Suess-corrected δ^13^C trend (Figure 4) can also be attributed to increased primary production. Due to the preferential utilization of ^12^C by phytoplankton, periods of low primary productivity will result in relatively low δ^13^C (Wu and others 2006). Conversely, periods of high primary productivity will lead to a depletion of ^12^C in the C pool and thus increased uptake of ^13^C (Meyers and Teranes 2001). Lastly, the obtained δ^13^C values (Figure 5) are typical of autotrophic lakes (> − 27‰) and consistent with expectations for Lake Victoria (Verburg 2007)*.* The lack of increasing δ^13^C in SC19 (Figure 5) may result from deeper sediment mixing (as suggested by the ^210^Pb profile) compared to SC12 due to the higher potential of trawling at the SC19 coring site (Witte and other 2012), which may have stimulated selective preservation of specific fractions of organic matter (Lehmann and others 2002).

### Food Web Responses to Eutrophication

Anthropogenic eutrophication led to major shifts in the algal (Figure 6) and cladoceran (Figure 7) assemblages of Mwanza Gulf. Despite low pigment preservation in tropical lakes (Buchaca and others 2019), pigments have been used to successfully reconstruct major shifts in primary production in Lake Victoria (Wienhues and others 2024). In Mwanza Gulf, pigment degradation remained relatively stable across the cores (CPI < 0.1; Figure 6). Thus, increased pigment deposition upcore likely reflects increased production rather than degradation.

Initial increases in total primary production around ~ 1920 (TChl) occurred gradually alongside increased nutrient loading (TP and TN). A doubling of phytoplankton production was similarly observed in northern inshore waters during this period (Mugidde 1993). The majority of sedimentary pigments increased gradually starting around ~ 1920, suggesting that all analyzed taxonomic groups benefited from increased nutrient availability. Furthermore, the high-resolution TChl values effectively captured the fluctuations in total primary production over time in more detail (Figure 4) than the pigment data. The phytoplankton assemblage remained dominated by diatoms as indicated by the continuous abundance of diatom-related pigments (diatoxanthin and diadinoxanthin), while the abundance of cyanobacteria pigment canthaxanthin indicates that cyanobacteria were at least seasonally abundant.

The subsequent assemblage shift around ~ 1990 CE (± 2 years) indicates increasing dominance of cyanobacteria-related pigments, whereas diatom- and dinoflagellate-related pigments remained stable or decreased (diatoxanthin, diadinoxanthin, dinoxanthin) and remained most likely abundant at low levels (Figure 6). The timing of this shift is consistent with phytoplankton monitoring data that indicate stabilization of phytoplankton abundance since the 1990s in the Mwanza Gulf (Franke and others 2023). Accordingly, diatoms and cyanobacteria have been the main phytoplankton groups in the Mwanza Gulf, whereas chlorophytes have remained very low or absent. Increased abundances of phytoplankton occurred despite relatively stable δ^15^N, suggesting that other factors (for example, temperature) may be influencing algal abundance in addition to increased nutrients.

Changes in the abundance and composition of primary producers did not coincide with the major shift in the cladoceran assemblage in ~ 1957 (± 5 years; Figure 7, S7). Despite the significant decline in cladocerans, the phytoplankton assemblage did not exhibit a corresponding increase in abundance, suggesting that reduced cladoceran grazing pressure did not contribute to the increase in phytoplankton. It is important to note that not all zooplankton taxa (for example, copepods) are preserved in lake sediments; thus, reductions in cladoceran abundances do not necessarily imply a reduction in overall zooplankton biomass. However, the decline in cladoceran abundance may be partially attributed to changes in the nutritional quality of the phytoplankton community (Cebrian and others 2009). In eutrophic lakes, zooplankton biomass can be uncorrelated with phytoplankton biomass (Yuan and Pollard 2018) in large part due to the proliferation of inedible phytoplankton, particularly cyanobacteria (Heathcote and others 2016). Cyanobacteria are poorly utilized by herbivorous zooplankton as a food source because they offer low nutritional value, pose toxicity risks, and have physical features that make them challenging to ingest (Vanni 1987; de Bernardi and Giussani 1990; Müller-Navarra and others 2000). Notably, *Bosmina longirostris* can be resistant to some toxins, allowing it to coexist with toxic cyanobacteria blooms (Adamczuk 2016), which may help explain its rising abundances at the turn of the century (Figure 7).

Abundances of all cladoceran taxa in Mwanza Gulf decreased substantially in ~ 1957 (± 5 years), possibly due to the cumulative impacts of lake-level rise and anthropogenic eutrophication. The parallel decline of both benthic chydorids and *Bosmina* (Figure 7) suggests that depth-associated changes in habitat conditions, such as a shift of the littoral zone or loss of macrophyte habitat, could have contributed to the observed collapse of the cladoceran community. Changes in lake water depth can strongly influence cladoceran assemblages by altering the extent of littoral and pelagic habitats (Nevalainen and others 2011). Benthic chydorids (for example, *Alona* and *Chydorus*), although capable of open water migration, commonly inhabit clear, shallow waters with high macrophyte cover in other eastern African lakes (Verschuren and others 2000). Comparatively, rising lake levels may have made planktonic *Bosmina* more vulnerable to predation by reducing refuge afforded by submerged open-water macrophytes (Iglesias and others 2007). Together with the higher water levels, enhanced algal production in the Mwanza Gulf (Figure 6) could have led to decreased water transparency (Verschuren and others 2002) and light availability for littoral macrophytes (Natugonza and others 2021). Turbid waters likely inhibited macrophyte establishment due to reduced light penetration and reduced suitable chydorid habitat (Whiteside and Swindoll 1988). Alternatively, benthic chydorids may have moved into the newly established and inundated littoral environment and not been recovered at the core site due to spatial heterogeneity of cladoceran assemblages (for example, Nevalainen 2011). Interestingly, this cladoceran response preceded the collapse of haplochromine cichlids in the 1980s (Figure 1) and is consistent with previous studies indicating higher nearshore and offshore abundances of cladocerans in the early twentieth century compared to present (Mwebaza-Ndawula 1994).

In addition to lake-level rise and eutrophication, interspecific competition as well as invertebrate and fish predation may have led to changes in the zooplankton assemblages of Lake Victoria over the past century (Black and Hairston 1988; Branstrator and others 2003). Discontinuous monitoring indicates that decreased cladoceran abundances in Mwanza Gulf were accompanied by increased cyclopoid copepod abundances, which are cladoceran predators (Wanink and others 2002). Copepods, which make up high fractions of the contemporary zooplankton biomass in Lake Victoria, may have outcompeted small cladocerans as lake conditions changed, possibly due to greater hypoxia tolerance (Vanderploeg and others 2009) or better predator avoidance (Semyalo and others 2009). Furthermore, previous cores from northern Lake Victoria displayed a substantial post-1960s increase in *Chaoborus* abundances that occurred alongside decreasing cladoceran abundances (Bridgeman 2001). We did not observe this pattern in our samples, which contained relatively few *Chaoborus* remains, likely due to not reaching the minimum threshold of remains for reliably estimating past abundance (Figure S8; Quinlan and Smol 2010). This limitation arose from our sampling strategy, which primarily targeted Cladocera rather than *Chaoborus*. Therefore, further efforts are necessary to elucidate the temporal patterns of *Chaoborus* abundance and the predation pressure exerted on Cladocera and copepods in Mwanza Gulf.

Following the surge in Nile perch abundance, the replacement of zooplanktivorous haplochromines by dagaa as the dominant pelagic zooplanktivore in the 1980s (Gophen and others 1995) could also have affected the zooplankton communities. Despite increased dagaa abundances, the overall biomass of zooplanktivores decreased, suggesting that overall predation pressures also decreased (Wanink and others 2002). Whereas some studies have speculated that the shift in zooplankton assemblage is attributable to increased dagaa abundances (van Zwieten and others 2016), our time series demonstrate that the collapse of cladocerans clearly predates changes in the fish community in Mwanza Gulf (Figure 7). However, as nearshore turbidity improved in the 1990s (Sitoki and others 2010), ongoing fishing pressures contributed to declines in Nile perch population and the recovery of haplochromines in the Mwanza Gulf (Witte and others 2000). Zooplanktivores made up only ~ 10–20% of the haplochromine community prior to their decline, but were one of the dominant trophic guilds to rapidly resurge in the 1990s, constituting ~ 80% of the community (Witte and others 2007). The concurrent increases in *Bosmina* (Figure 7) suggest that the recovery of haplochromines may have released some of the controls (for example, *Chaoborus* or copepod predation) limiting *Bosmina* abundance.

## Conclusions

This study demonstrates that anthropogenic eutrophication profoundly altered planktonic community structure of Mwanza Gulf. Nutrient increases beginning in ~ 1920 promoted higher algal abundances, particularly of cyanobacteria, which in combination with rising lake-levels in the ~ 1960s led to habitat alterations that triggered the decline of both benthic and pelagic cladocerans. The collapse of biomass and species diversity of endemic haplochromines and subsequent recovery of their biomass may have further impacted planktonic *Bosmina* through changes in predation pressure. The lack of a compensatory response in the phytoplankton community, whereby algal biomass increases with decreasing cladoceran abundance, suggests weak top–down control of the algal biomass by cladoceran grazing pressure. Altogether, this study helps unravel additional insights to the food web dynamics underlying anthropogenic eutrophication and the loss of fish stocks in Lake Victoria.

### Supplementary Information

Below is the link to the electronic supplementary material.

Supplementary file1 (DOCX 1699 KB)

## Data Availability

All data are available in Zenodo: https://doi.org/10.5281/zenodo.10870087.
