# Supplementary material for: Anthropogenic Eutrophication Drives Major Food Web Changes in Mwanza Gulf, Lake Victoria
Source: Ecosystems. 2024 May 13;27(4):577–91. doi: 10.1007/s10021-024-00908-x (PMC11182866; doi:10.1007/s10021-024-00908-x)
Supplement: Supplementary file 1 — Supplementary file1 (DOCX 1699 KB) [file 10021_2024_908_MOESM1_ESM.docx]

**SUPPLEMENTAL MATERIAL**

King^*^ L, Wienhues^*^ G, Misra P, Tylmann W, Lami A, Bernasconi SM, Jaggi M, Courtney-Mustaphi C, Muschick M, Ngoepe N, Mwaiko S, Kishe MA, Cohen A, Heiri O, Seehausen O, Vogel H, Grosjean M, Matthews B. Anthropogenic eutrophication drives major food web changes in Mwanza Gulf, Lake Victoria. Ecosystems.

**
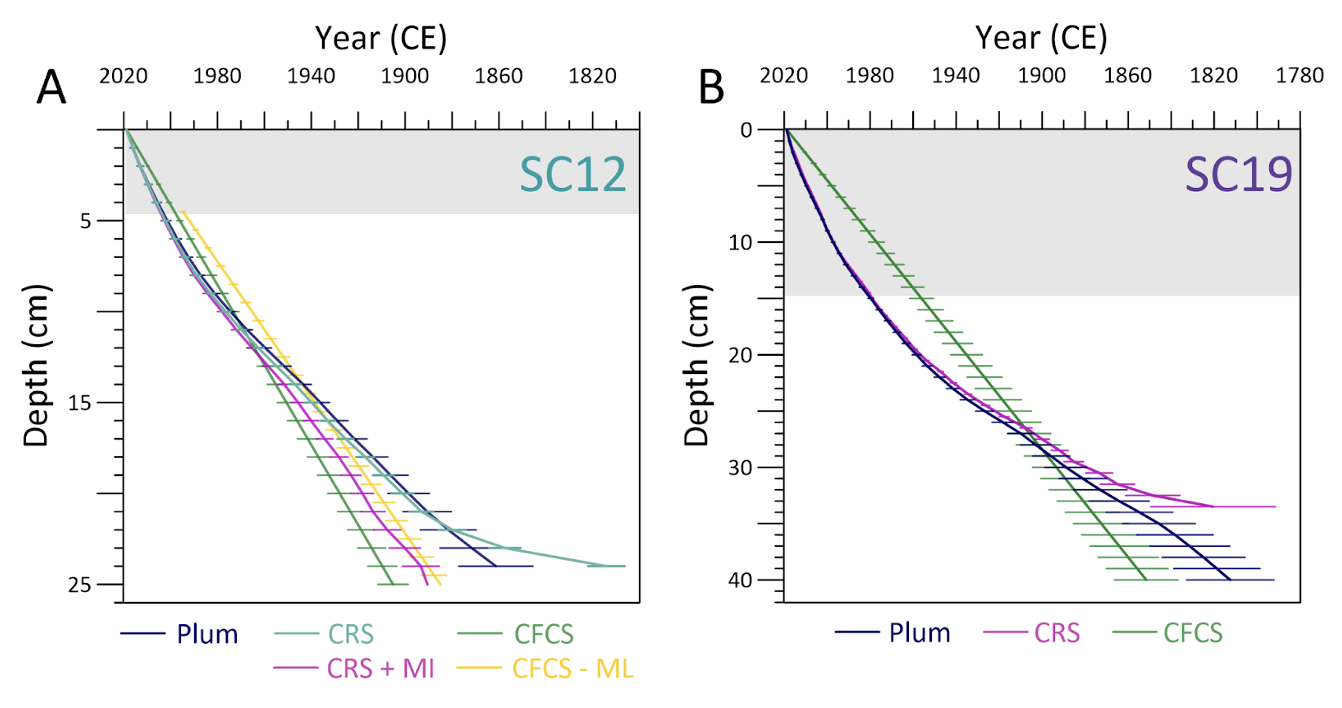
**

**Figure S1.** Alternative age-depth models considered for SC12 (A) and SC19 (B), including the Constant Rate of Supply (CRS), missing inventory corrected Constant Rate of Supply (CRS + MI), Constant Flux:Constant Sedimentation (CFCS), Constant Flux:Constant Sedimentation excluding the turbated layer (CFCS - ML), and Bayesian *plum* models. Gray areas indicate core layers with turbated sediment.


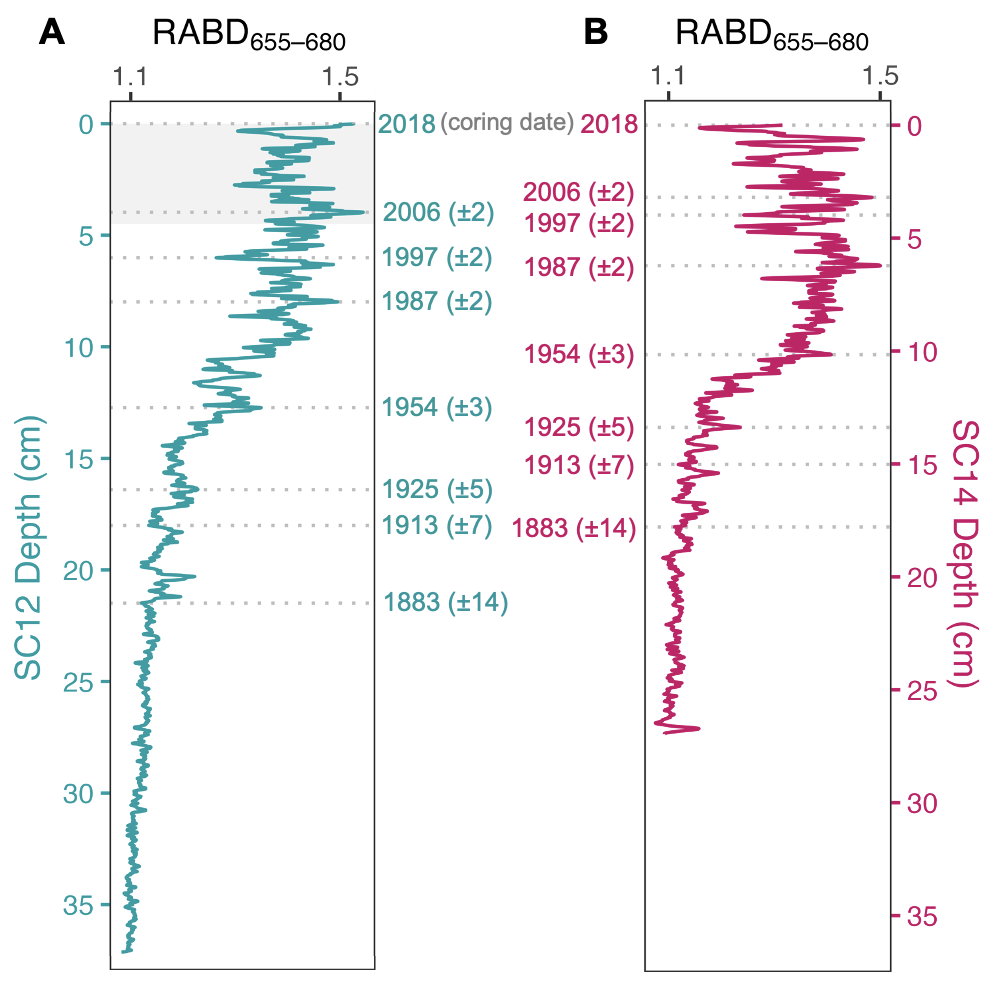


**Figure S2.** Correlation points between hyperspectral-inferred RABD_655-680_ profiles used to apply the SC12 (A) chronology to its paired core, SC14 (B), with ages linearly interpolated between correlation points.


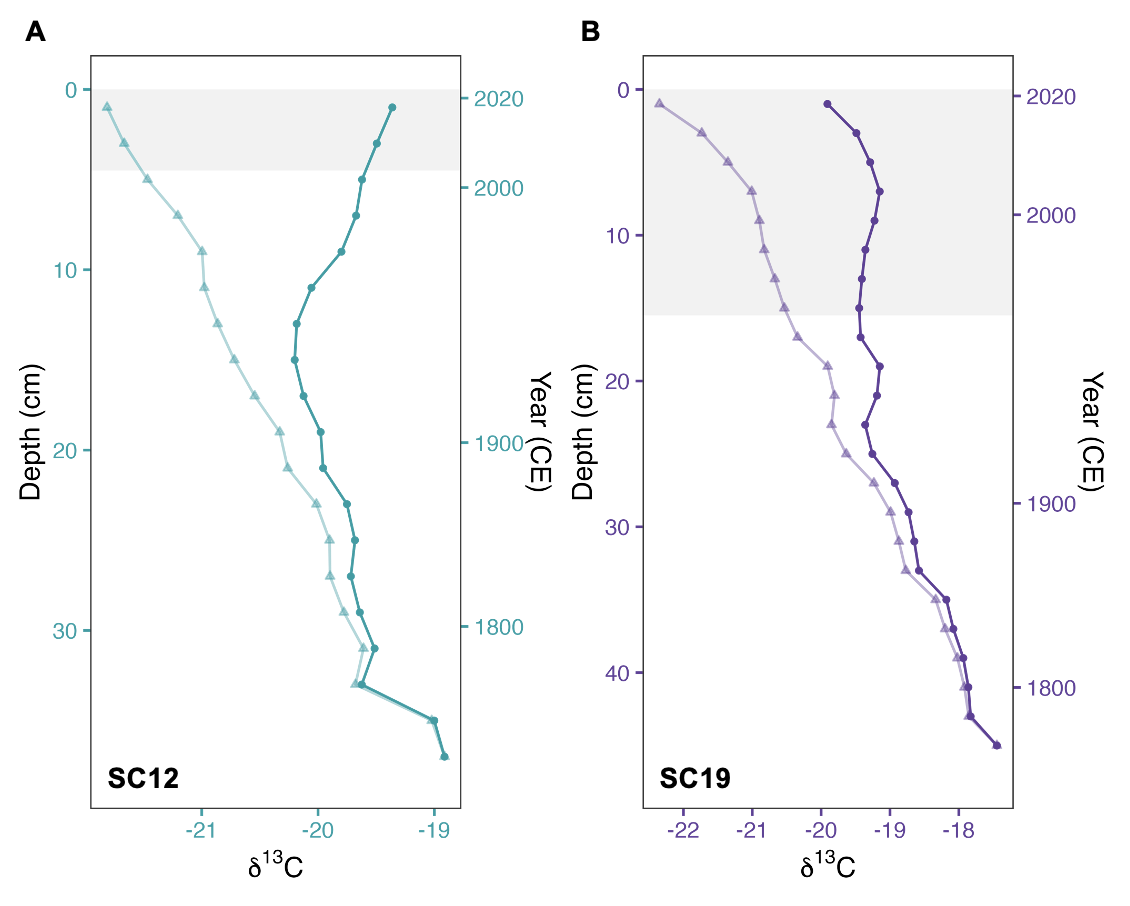


**Figure S3.** Suess-corrected δ^13^C values (dark circles), following Verburg (2007), compared with original δ^13^C signatures (light triangles) of SC12 (A) and SC19 (B).


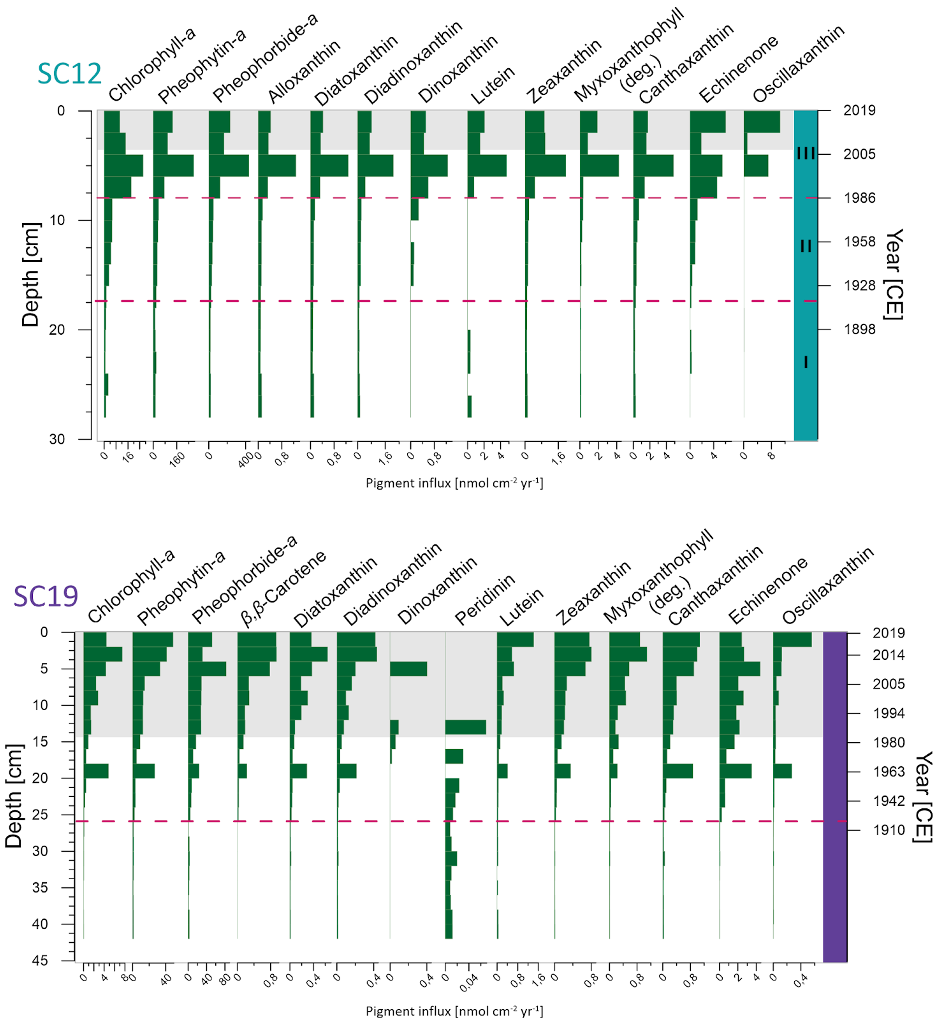
**Figure S4.** Pigment influxes for the cores SC12 and SC19.

**
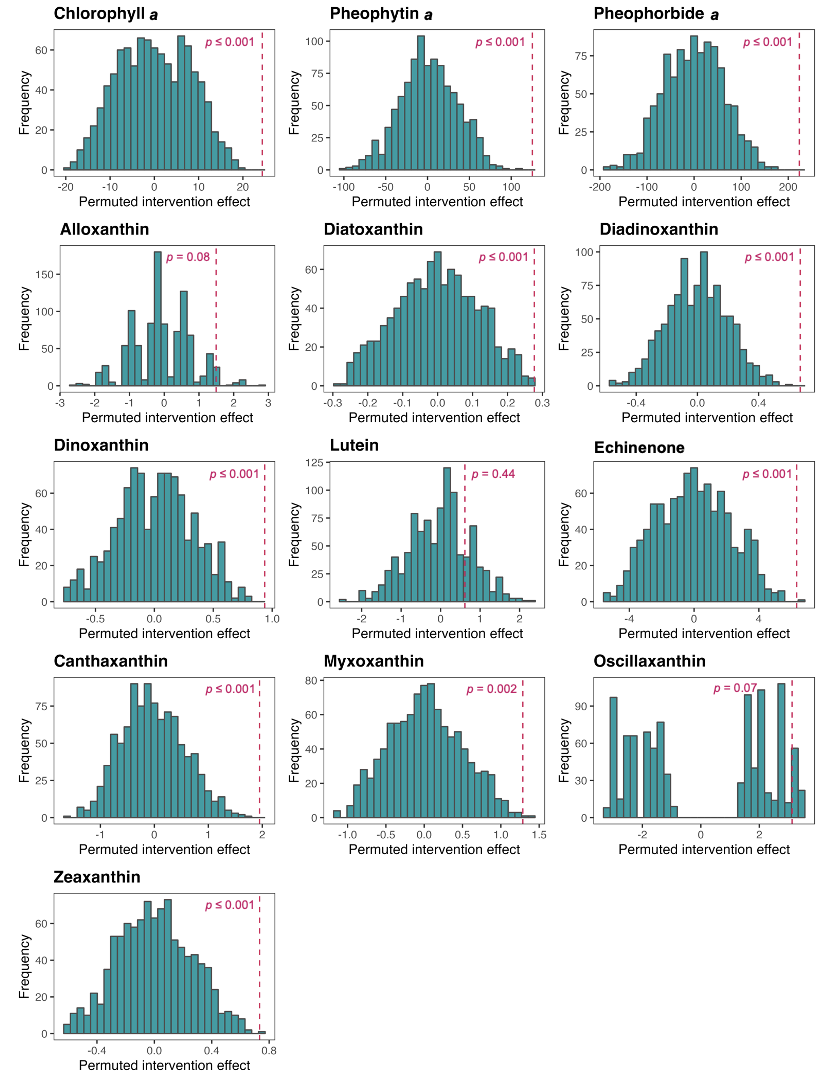
**

**Figure S5.** Intervention analysis of SC12 pigment concentrations using 18 cm core depth as the intervention point to represent 1920 CE. Dashed lines indicate the observed intervention effect.


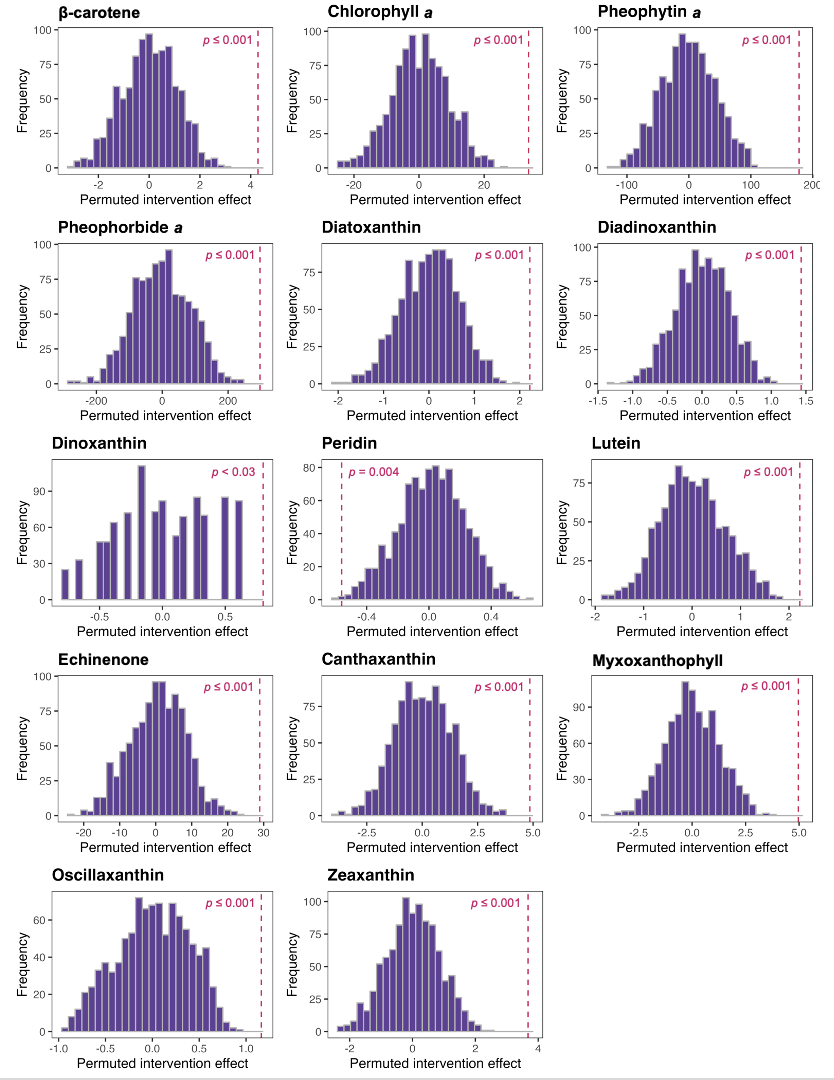


**Figure S6.** Intervention analysis of SC19 pigment concentrations using 26 cm core depth as the intervention point to represent 1920 CE. Dashed lines indicate the observed intervention effect.


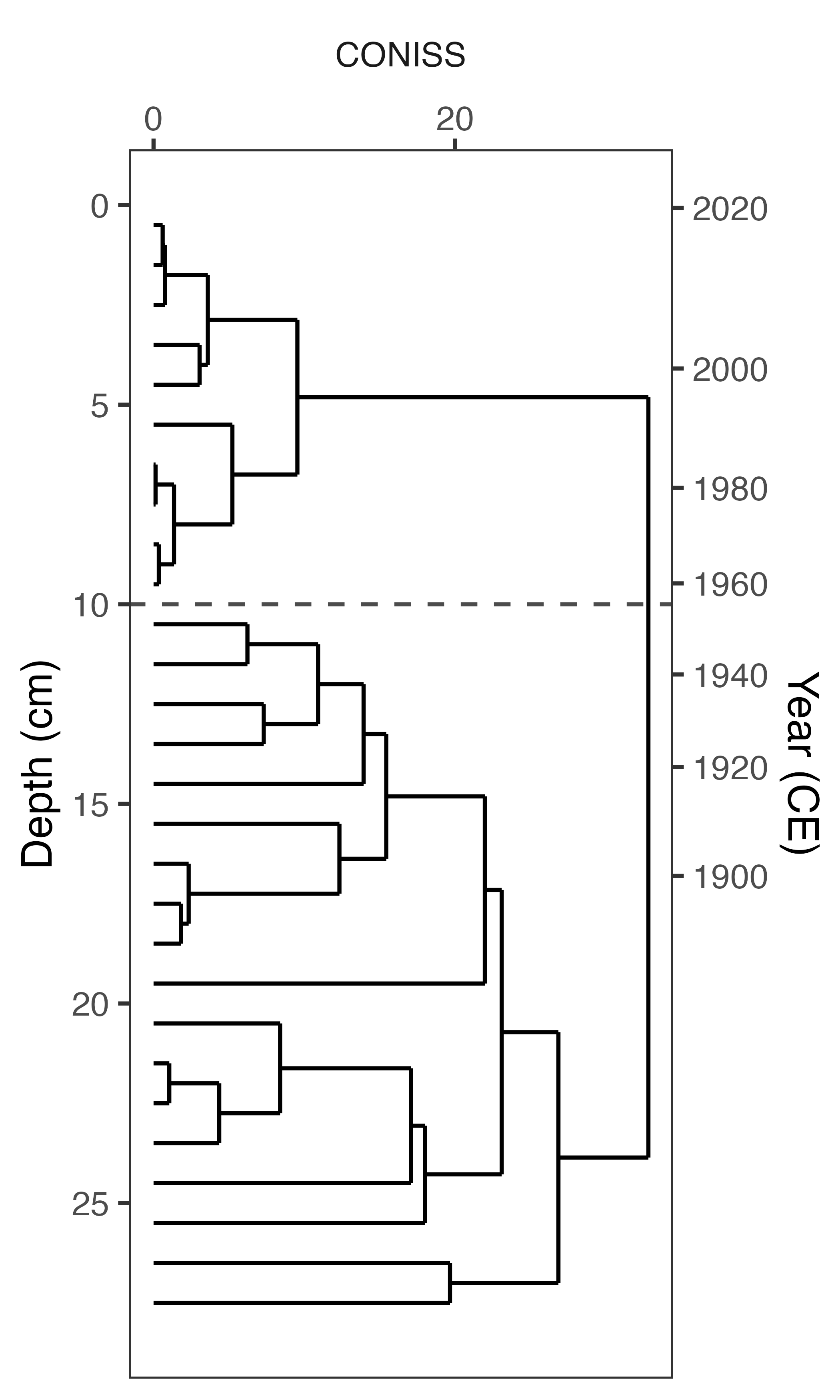


**Figure S7.** CONISS dendrogram for SC14 with dashed line indicating distinct cladoceran assemblages.


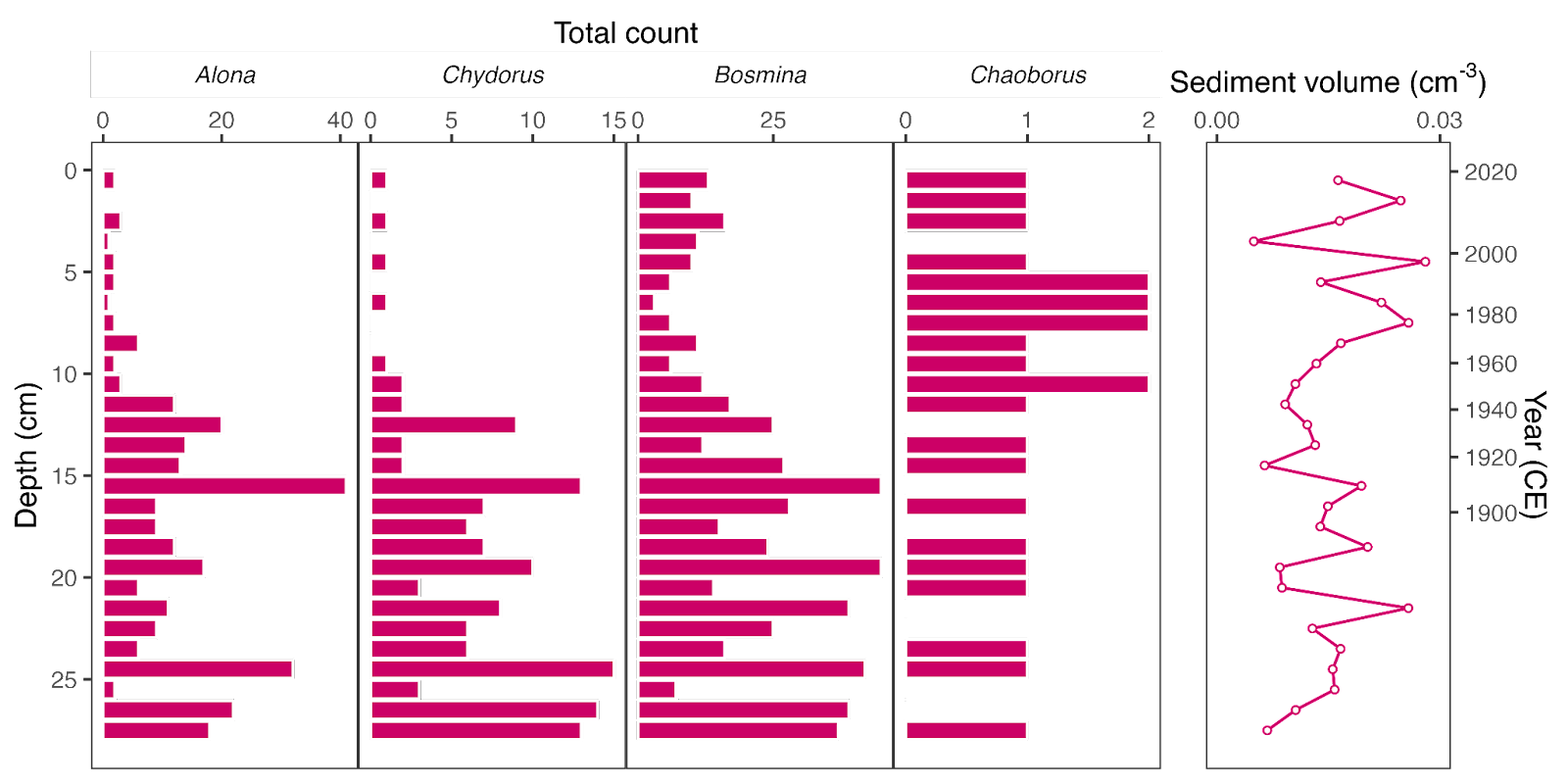


**Figure S8.** Total counts of Cladocera and *Chaoborus* individuals in SC14, determined by the minimum number of individuals possible given the most abundant body part of each taxon, and the total volume of sediment examined in each subsample.


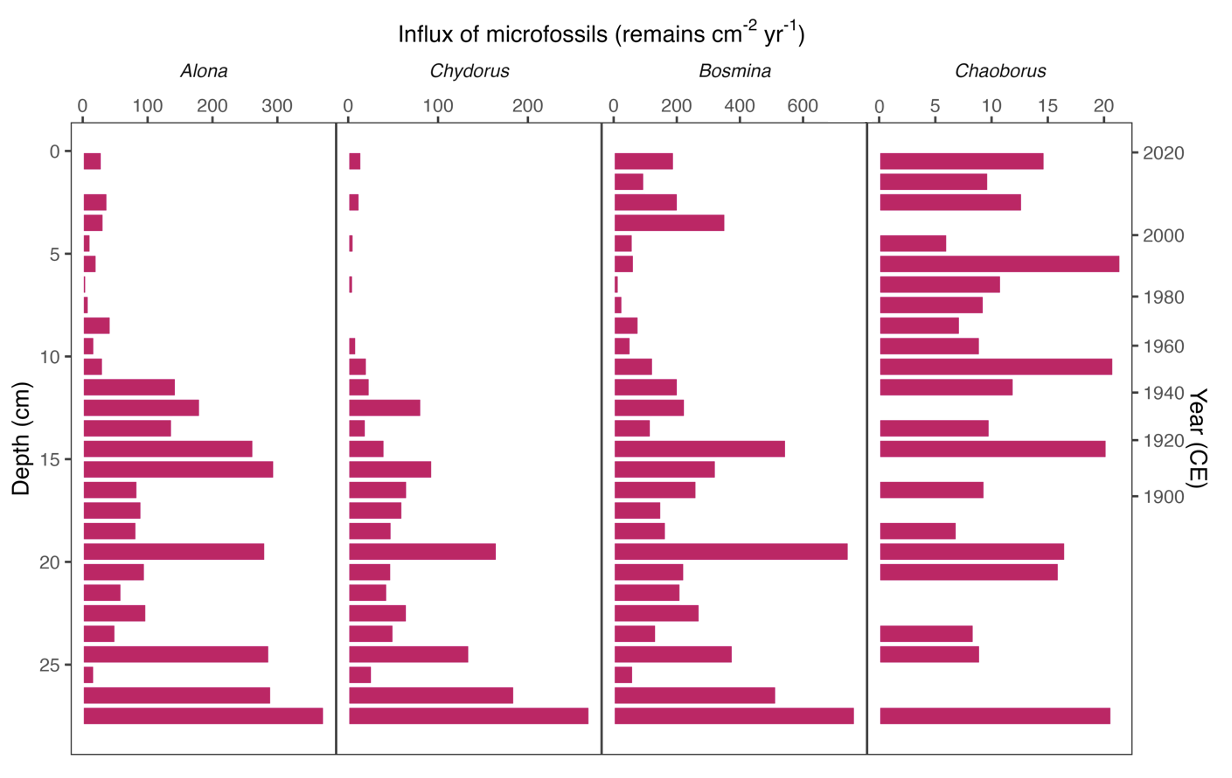


**Figure S9.** Influx of Cladocera and *Chaoborus* microfossils counted throughout SC14.


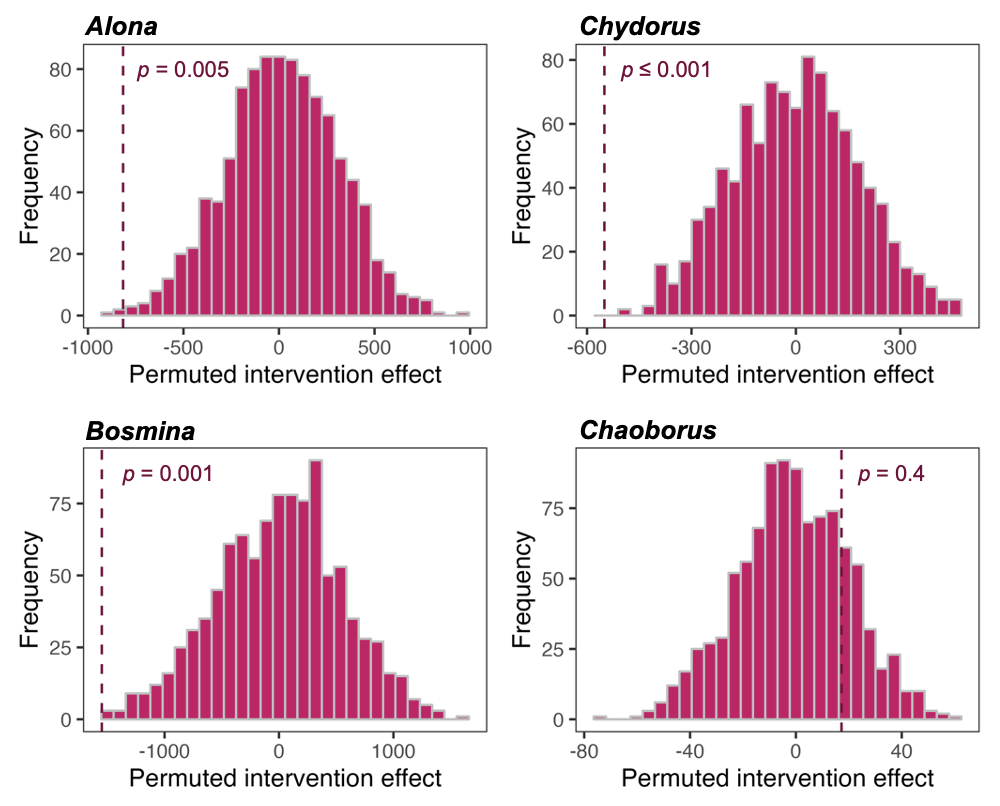


**Figure S10.** Intervention analysis of SC14 Cladocera and *Chaoborus* microfossil concentrations using 14 cm core depth as the intervention point to represent 1920 CE. Dashed lines indicate the observed intervention effect.

**Table S1.** Summary of SC12 core collected in October 2018 subsample data incorporated into the *plum* model, using a supported ^210^Pb concentration of 48.3 (± 4.7) Bq kg^-1^ (mean activity from the lowermost part of the core profile; excluding the bottom three samples).

| **Lab ID** | **Depth interval**  **(cm)** | **Density**  **(g cm^-3^)** | **Total ^210^Pb**  **(Bq kg^-1^)** | **sd(^210^Pb)** |
| --- | --- | --- | --- | --- |
| LV_SC12_1 | 0–1 | 0.006274 | 342.1 | 10.9 |
| LV_SC12_2 | 1–2 | 0.012528 | 358.3 | 11.1 |
| LV_SC12_3 | 2–3 | 0.012311 | 360.7 | 11.4 |
| LV_SC12_4 | 3–4 | 0.011631 | 363.4 | 16.0 |
| LV_SC12_5 | 4–5 | 0.011135 | 358.1 | 20.7 |
| LV_SC12_6 | 5–6 | 0.011319 | 324.8 | 17.4 |
| LV_SC12_7 | 6–7 | 0.011559 | 305.5 | 14.2 |
| LV_SC12_8 | 7–8 | 0.011802 | 293.4 | 11.6 |
| LV_SC12_9 | 8–9 | 0.011614 | 287.4 | 9.1 |
| LV_SC12_10 | 9–10 | 0.012136 | 259.3 | 8.4 |
| LV_SC12_11 | 10–11 | 0.012193 | 225.1 | 7.7 |
| LV_SC12_12 | 11–12 | 0.011898 | 210.5 | 7.5 |
| LV_SC12_13 | 12–13 | 0.010945 | 187.8 | 7.3 |
| LV_SC12_14 | 13–14 | 0.010721 | 161 | 6.5 |
| LV_SC12_15 | 14–15 | 0.010842 | 126.1 | 5.6 |
| LV_SC12_16 | 15–16 | 0.010212 | 111.5 | 5.2 |
| LV_SC12_17 | 16–17 | 0.010161 | 102.8 | 4.8 |
| LV_SC12_18 | 17–18 | 0.010338 | 95 | 4.5 |
| LV_SC12_19 | 18–19 | 0.00994 | 81.2 | 4.2 |
| LV_SC12_20 | 19–20 | 0.009691 | 76.7 | 3.9 |
| LV_SC12_21 | 20–21 | 0.009101 | 70.2 | 3.6 |
| LV_SC12_22 | 21–22 | 0.009627 | 71.5 | 3.8 |
| LV_SC12_23 | 22–23 | 0.00985 | 72.3 | 3.9 |
| LV_SC12_24 | 23–24 | 0.009292 | 65.8 | 3.5 |
| LV_SC12_25 | 24–25 | 0.009395 | 54.3 | 3.1 |
| LV_SC12_26 | 25–26 | 0.00956 | 52 | 3.1 |
| LV_SC12_27 | 26–27 | 0.009661 | 50.6 | 3.1 |
| LV_SC12_28 | 27–28 | 0.009665 | 49.4 | 3.1 |
| LV_SC12_29 | 28–29 | 0.009642 | 48.7 | 3.1 |
| LV_SC12_31 | 30–31 | 0.019163 | 46.5 | 3.2 |
| LV_SC12_33 | 32–33 | 0.018397 | 38.7 | 2.8 |
| LV_SC12_35 | 34–35 | 0.017184 | 46.1 | 3.3 |

**Table S2.** Summary of SC19 core collected in October 2018 subsample data incorporated into the Bayesian *plum* model, using a supported ^210^Pb concentration of 52.6 (± 1.3) Bq kg^-1^ (mean activity from the lowermost part of the core profile).

| **LabID** | **Depth interval**  **(cm)** | **Density**  **(g cm^-3^)** | **^210^Pb**  **(Bq kg^-1^)** | **sd(^210^Pb)** |
| --- | --- | --- | --- | --- |
| LV_SC19_1 | 0–1 | 0.002312 | 309.6 | 11.1 |
| LV_SC19_2 | 1–2 | 0.004932 | 268.4 | 9.4 |
| LV_SC19_3 | 2–3 | 0.004925 | 335 | 11.2 |
| LV_SC19_4 | 3–4 | 0.004454 | 351.2 | 10.9 |
| LV_SC19_5 | 4–5 | 0.004153 | 363.5 | 11.4 |
| LV_SC19_6 | 5–6 | 0.004037 | 397.9 | 12.8 |
| LV_SC19_7 | 6–7 | 0.004065 | 354.6 | 11.8 |
| LV_SC19_8 | 7–8 | 0.004268 | 329.5 | 11.1 |
| LV_SC19_9 | 8–9 | 0.004360 | 267.2 | 8.9 |
| LV_SC19_10 | 9–10 | 0.004476 | 296.4 | 9.9 |
| LV_SC19_11 | 10–11 | 0.004745 | 260.9 | 9.0 |
| LV_SC19_12 | 11–12 | 0.004810 | 269.3 | 9.3 |
| LV_SC19_13 | 12–13 | 0.004654 | 289.6 | 10.4 |
| LV_SC19_14 | 13–14 | 0.004512 | 271.3 | 9.5 |
| LV_SC19_15 | 14–15 | 0.004422 | 276.4 | 9.7 |
| LV_SC19_16 | 15–16 | 0.004430 | 228 | 8.4 |
| LV_SC19_17 | 16–17 | 0.004552 | 214 | 7.9 |
| LV_SC19_18 | 17–18 | 0.004730 | 201.2 | 7.5 |
| LV_SC19_19 | 18–19 | 0.004506 | 185.7 | 7.0 |
| LV_SC19_20 | 19–20 | 0.004399 | 191.3 | 7.3 |
| LV_SC19_21 | 20–21 | 0.004544 | 163.9 | 6.4 |
| LV_SC19_22 | 21–22 | 0.004559 | 170.2 | 6.7 |
| LV_SC19_23 | 22–23 | 0.004244 | 158 | 6.5 |
| LV_SC19_24 | 23–24 | 0.004101 | 154.5 | 6.4 |
| LV_SC19_25 | 24–25 | 0.004056 | 147.4 | 6.1 |
| LV_SC19_26 | 25–26 | 0.004183 | 130.5 | 5.7 |
| LV_SC19_27 | 26–27 | 0.004507 | 107.2 | 4.9 |
| LV_SC19_28 | 27–28 | 0.004856 | 83.2 | 4.2 |
| LV_SC19_29 | 28–29 | 0.005047 | 78.9 | 4.0 |
| LV_SC19_30 | 29–30 | 0.005069 | 69.3 | 3.7 |
| LV_SC19_31 | 30–31 | 0.005268 | 75.4 | 4.1 |
| LV_SC19_32 | 31–32 | 0.005371 | 64.4 | 3.5 |
| LV_SC19_33 | 32–33 | 0.005060 | 68.3 | 3.9 |
| LV_SC19_34 | 33–34 | 0.00498 | 66.3 | 3.7 |
| LV_SC19_35 | 34–35 | 0.005034 | 62.5 | 3.6 |
| LV_SC19_36 | 35–36 | 0.005083 | 53.9 | 3.2 |
| LV_SC19_37 | 36–37 | 0.005179 | 51.9 | 3.1 |
| LV_SC19_38 | 37–38 | 0.005299 | 53.4 | 3.2 |
| LV_SC19_39 | 38–39 | 0.005314 | 50.4 | 3.3 |
| LV_SC19_40 | 39–40 | 0.005330 | 52.5 | 3.3 |
| LV_SC19_41 | 40–41 | 0.005476 | 53.4 | 3.5 |

**Table S3.** Randomized intervention analysis results of photosynthetic pigments.

|  | **SC12** | | | **SC19** | | |
| --- | --- | --- | --- | --- | --- | --- |
| **Pigment** | **Pre-1920** | **Post-1920** | **p-value** | **Pre-1920** | **Post-1920** | **p-value** |
| *β,β*-carotene | N/A | N/A | N/A | 0 | 3.96 | ≤0.001 |
| Chlorophyll *a* | 6.63 | 31.01 | ≤0.001 | 3.63 | 34.16 | ≤0.001 |
| Pheophytin *a* | 90.46 | 215.72 | ≤0.001 | 77.45 | 252.7 | ≤0.001 |
| Pheophorbide *a* | 103.17 | 326.73 | ≤0.001 | 135.46 | 451.66 | ≤0.001 |
| Alloxanthin | 1 | 2.49 | 0.08 | N/A | N/A | N/A |
| Diatoxanthin | 0.58 | 0.86 | ≤0.001 | 0.91 | 3.12 | ≤0.001 |
| Diadinoxanthin | 0.7 | 1.37 | ≤0.001 | 0.76 | 1.99 | ≤0.001 |
| Dinoxanthin | 0 | 0.94 | ≤0.001 | 0 | 0.62 | 0.03 |
| Peridinin | N/A | N/A | N/A | 0.78 | 0.44 | 0.004 |
| Lutein | 1.04 | 1.67 | 0.44 | 2.3 | 4.21 | ≤0.001 |
| Echinenone | 0.59 | 6.93 | ≤0.001 | 3.53 | 35.11 | ≤0.001 |
| Canthaxanthin | 1.51 | 3.46 | ≤0.001 | 2.06 | 6.67 | ≤0.001 |
| Myxoxanthophyll | 0.52 | 1.81 | 0.002 | 0.71 | 5.1 | ≤0.001 |
| Oscillaxanthin | 0.18 | 3.3 | 0.07 | 0.21 | 1.29 | ≤0.001 |
| Zeaxanthin | 0.65 | 1.39 | ≤0.001 | 0.66 | 4.14 | ≤0.001 |

**Table S4.** Randomized intervention analysis results of *Chaoborus* and Cladocera taxa.

|  | **SC14** | | |
| --- | --- | --- | --- |
| **Zooplankton** | **Pre-1920** | **Post-1920** | **p-value** |
| *Alona* | 1219 | 403 | 0.005 |
| *Chydorus* | 663 | 113 | ≤0.001 |
| *Bosmina* | 2448 | 899 | 0.001 |
| *Chaoborus* | 55 | 73 | 0.4 |
